# Experimental Assessment of Two Non-Contrast MRI Sequences Used for Computational Fluid Dynamics: Investigation of Consistency Between Techniques

**DOI:** 10.1007/s13239-020-00473-z

**Published:** 2020-07-01

**Authors:** C. J. MacDonald, R. Hellmuth, L. Priba, E. Murphy, S. Gandy, S. Matthew, R. Ross, J. G. Houston

**Affiliations:** 1 Imaging and Technology, University of Dundee, Dundee, UK; 2 Vascular Flow Technologies LTD, Dundee, UK; 3 Medical Physics, NHS Tayside, Dundee, UK; 4 Vascular Laboratory, NHS Tayside, Dundee, UK; 5 Molecular and Clinical Medicine, School of Medicine, University of Dundee, Dundee, UK

**Keywords:** Arterio-venous fistula, Magnetic resonance imaging, Computational fluid dynamics

## Abstract

**Purpose:**

Recent studies have noted a degree of variance between the geometries segmented by different groups from 3D medical images that are used in computational fluid dynamics (CFD) simulations of patient-specific cardiovascular systems. The aim of this study was to determine if the applied sequence of magnetic resonance imaging (MRI) also introduced observable variance in CFD results.

**Methods:**

Using a series of phantoms MR images of vessels of known diameter were assessed for the time-of-flight and multi-echo data image combination sequences. Following this, patient images of arterio-venous fistulas were acquired using the same sequences. Comparisons of geometry were made using the phantom and patient images, and of wall shear stress quantities using the CFD results from the patient images.

**Results:**

Phantom images showed deviations in diameter between 0 and 15% between the sequences, depending on vessel diameter. Patient images showed different geometrical features such as narrowings that were not present on both sequences. Distributions of wall shear stress (WSS) quantities differed from simulations between the geometries obtained from the sequences.

**Conclusion:**

In conclusion, choosing different MRI sequences resulted in slightly different geometries of the same anatomy, which led to compounded errors in WSS quantities from CFD simulation.

**Electronic supplementary material:**

The online version of this article (10.1007/s13239-020-00473-z) contains supplementary material, which is available to authorized users.

## Introduction

Computational Fluid Dynamics (CFD) is a well-known tool in engineering, used to assess the flow of fluids *in silico*. This computational method is applied in cardiovascular engineering to predict and analyze blood flow patterns in complex situations, such as cerebral aneurysms, valve prosthesis, stented vessels, and arteriovenous fistulae (AVFs).^16^ Commonly, these studies are performed using 3D geometries segmented from medical images, such as computed tomography (CT) and magnetic resonance imaging (MRI), with or without contrast agent. Certain patient groups cannot receive Gd-based contrast agents commonly used for MRI,^1^ so non-contrast (NCE) MRI sequences are used to image blood vessels in these cases. Multiple NCE sequences exist, including the well-known time of flight (ToF), TrueFISP, and the multi echo data image combination (MEDIC) sequence which has been shown to depict vessel diameters in agreement with ultrasound when used in the upper extremity.^13^

The accuracy of CFD in predicting flow dynamics depends on a series of factors including the boundary conditions, turbulence modeling, meshing techniques, researcher experience, and geometrical accuracy. Recently, the American Food and Drug Administration (FDA) has begun to consider a good practice to use computational models in the design process of medical devices. The FDA conducted a multi-center study to compare CFD results to an experimental model, and observed a large degree of variability between research groups.^21^ Another large study conducted by The International Aneurysm Challenged observed wide variability in CFD results between researchers.^26^ Similarly, variations in results have been observed when segmentations from MRI images and CT images are compared, and when imaging of the same participant is undertaken at different time points^23^ suggesting that deviations in geometry are an important factor in CFD variation. It is possible different MRI sequences can introduce similar variability into blood vessel segmentation, and, consequently, the CFD analysis.

Used as a vascular access for hemodialysis,^6^ the autologous AVF is a non-physiological anatomy formed by creating an anastomosis between an artery and vein, which produces a blood-flow in the vein which would never occur naturally. However, AVFs suffer from failure rates of around 30–40% at one year,^2^ typically due to stenosis secondary to occlusion. AVF failure has associated cost and morbidity, and can significantly disrupt a patient’s life. The AVF presents an interesting case for CFD studies due to intertwined morphological and hemodynamic changes, which occur after its creation, which ultimately affect its clinical usability. Wall shear stress (WSS) patterns have been identified as having a significant effect on the development of stenoses in AVFs, and has been studied extensively using CFD. In these studies a number of different MRI sequences have been used in geometry acquisition.^7^,^10^,^11^,^20^

The aim of this study was to assess whether ToF and MEDIC MRI produce similar depiction of geometry, and similar results from corresponding CFD simulations. Specifically, we hypothesized that since MEDIC has been shown to agree with US measurements of peripheral vessels, and ToF to underestimate their size, WSS quantities from ToF would be overestimated in CFD simulation. In order to assess the differences introduced by changing MRI sequence, a series of phantoms simulating morphological features of the AVF were scanned. Using these phantoms, the objectives were to explore the effect of vessel diameter, flow velocity in the vessel, and flow direction relative to the imaging plane on the geometry of segmented models used for CFD analysis. To follow up, WSS quantities were obtained from patient-specific CFD simulations using geometries obtained from the two MRI sequences.

## Methods

Briefly, this study examined differences in geometric and simulated WSS quantities between ToF and MEDIC MRI, and was achieved in the following steps. A series of phantoms were used to obtain MR images of vessels of known diameter, allowing observation of the effect of changing pulse sequence parameters, vessel size, vessel flow, and vessel orientation on geometry measurements. WSS quantities were determined in order to give a baseline error value. Next, patient images were acquired from the two sequences and segmented to generate WSS quantities from CFD. WSS quantities from the two sequences were compared to determine whether changing the MRI sequence had measurable effects on CFD simulations.

### Phantom Preparation

Two phantom setups were manufactured, a straight tube aligned with the MRI scanner z-axis, and a loop swing. The first setup consisted of an open acrylic box, with a couple of supporting structures glued to a plastic tube with its inlet and outlet points on the opposite walls of the box. Three phantoms of this type were manufactured, with internal tube diameters (*D*) 2, 3 and 5 mm and 1-1.6 mm wall thickness. The inlet of the tubing on one side wall of the box was connected to a water source and a pulsatile flow pump (Cole Parmer, Masterflex Digital Pump System, Germany). Each phantom was imaged with water flowing at flow rates (*Q*) which maintained a flow velocity of 0.5 or 1.0 m s^−1^ in the phantom. With water density *ρ* = 1 × 10^3^ kg m^*−*3^ and dynamic viscosity *µ* = 1 × 10^*−*3^ Pa s, the Reynolds number $Re = \frac{4}{\pi }\frac{\rho Q}{\mu D}$ of the flow in the phantoms ranged from 2100–10,600, indicating that the flow conditions were either transitional or turbulent.

The second phantom setup consisted of an open acrylic box with both inlet and outlet points positioned on the same wall. In this case, three plastic cylinders were glued into the phantom body, two of which acted as supporting structures, and the most distal one as a wrapping post (curve-*H*). This setup was then repeated with the tubing wrapped around the center cylinders (curve-*L*). The effect of this was to decrease the curvature of the phantom vessel. In other words, phantom curve-*H* had a higher curvature than phantom curve-*L*. All phantoms were filled with a small amount of water in order to prime the scanner. All phantoms were placed into the scanner bore with the center of the phantom aligned with the scanner isocenter. Images of the phantoms can be seen in the supplementary material (sup. Figs. 1 and 2).

### Patient Population

Four patients with end-stage renal failure who had been referred for AVF creation surgery at our institution were recruited into this study as part of the ReDVA project (www.redva.eu), a multicenter study aiming to assess longitudinal changes in AVFs using CFD . All patients provided informed written consent, and ethical approval was obtained. Two of the patients were indicated for brachio-cephalic AVF creation (AV1, AV3) and two were indicated for radio-cephalic AVF creation (AV2, AV4). Post AVF surgery, all patients underwent an MRI surveillance session, 17–26 days after surgery.

All patients were placed head first and supine into the bore with their arms relaxed by their side. An 8-channel phased array RF coil was placed around the arm of interest. The patient was positioned slightly off-axis in relation to the scanner bore, in order to ensure that the arm (anatomical area of interest) was as near as possible to the isocenter of the magnet. The site of the anastomosis was identified by palpable thrill and was marked by positioning a cod liver oil capsule on the skin adjacent to the site, which can be seen in the supplementary material (sup. Fig. 3).

### MR Imaging

All images were acquired on a 3.0 T PrismaFIT scanner (SIEMENS, Erlangen, Germany) with an 8-channel small flexible array coil. A 2D gradient echo localizer sequence was used for initial visualization of the area of interest. Following this, a 2D ToF MR sequence was applied in an axial oblique orientation and region coverage was maintained at approximately 10 cm to 15 cm. Imaging parameters used were TR/TE: 14/5.8 ms, FA 18°, slice thickness: 1.5 mm, FOV: 140 mm, matrix: 512 × 512 px (no interpolation), and receiver bandwidth: 165 Hz px^*−*1^. This was followed by a 3D T2* MEDIC sequence (TR/TE: 29/16 ms, FA: 30°, slice thickness: 1.06 mm (176 slices in the imaging block), FoV: 136 mm, matrix: 512 × 512 px (no interpolation), and receiver bandwidth: 160 Hz px^−1^) over the same area. The MRI sequences had similar voxel sizes, with vox_*ToF*_ = 0.273 mm and vox_*MEDIC*_ = 0.266 mm.

Patients had one additional sequence added to their protocol in order to obtain flow velocity measurements. A retrospectively ECG gated 2D phase-contrast (PC) MRI was performed, both proximally and distally (approx. 5 cm) to the anastomosis, in order to measure the through plane flow rates at each branch of the AVF. Imaging parameters used were TR/TE: 99.7/7.62 ms, FA: 20°, FOV 100 mm, matrix 192 × 115, receiver bandwidth: 440 Hz/pixel, VENC: 10–250 cm s^*−*1^ (depending on whether artery or vein) and 16–64 temporal phases over the cardiac cycle. Velocity wave-forms for the blood flow were produced by semi-automated segmentation using Segment (Medviso, Switzerland).

### Vessel Segmentation

All MEDIC and ToF images were segmented using the sweeping method available on SimVascular (Stanford University, CA, USA).^25^ Nodes of 3D splines were manually positioned near the center of the vessel for working as sweeping pathlines. Next, the 3D scans were interpolated onto a sequence of planes perpendicular to the pathlines, where 2D closed-loop splines segmenting the vessel lumen were obtained. Finally, the closed-loop spline segmentations were lofted together to form the 3D tubular models with smooth transition between the segmented planes. These models were exported as finely-spaced stereolithography (STL) files for geometry analysis and CFD meshing.

### Geometry Analysis

To compare geometric features (primarily vessel diameter and area) between the MRI sequences it was necessary to define an origin point shared between the images. For the phantom models the origin point was defined as the first segment of vessel to enter the phantom box area. For the patient models the origin point was defined as the point at the anastomosis where the vein centerline intersects with the artery centerline.

Vessel centrelines in the form of splines were extracted using the maximum inscribed sphere radius method.^3^ The distance along the centerlines to the defined origin point *s* was defined as a topological 1D coordinate system to locate and compare the geometrical features of the vessel. Thus the Cartesian position of any point of the centerline splines was found from the distance along the centerline as ***γ***(*s*) = (*x*(*s*)*, y*(*s*)*, z*(*s*)), where the distance coordinate *s* was obtained from a line integral of ***γ*** using any arbitrary parametric coordinate. The vessel cross-sectional area *A* = *A*(*s*) was obtained by integrating slices of the geometrical models perpendicular to the centerline.

### Computational Fluid Dynamics

The CFD meshing and processing were both performed with HELYX v.2.5 using the STL files obtained from the patient images. A hexahedra-dominant octree algorithm was used for meshing, which is the native mesh method in the OpenFOAM package. This method generates a surface mesh with defined cell spacing, totally independent of the quality of the STL surface obtained from the segmentation. Furthermore, this method minimises the formation of non-orthogonal cell surfaces, which introduce integration errors in the finite volume method (FVM). For the patient models, the mesh surface was divided by four patches: wall, proximal artery, distal artery, and proximal vein; at which the boundary conditions were applied. Most cells had sides of 250 µm, and an inflation prism layer of five cells from 50 to 125 µm covered the wall patch. The walls were considered rigid, and the boundary conditions of all exits of the fistula were specified according to the patient-specific flow rate pulses obtained from the PC-MRI scans. The flow rate time series of the proximal artery and vein were obtained by integrating the same 2D PCMRI cross-sectional plane. These flow rates were used to apply velocity values in a paraboloid distribution to both the proximal artery and proximal vein caps, whilst the distal artery velocity boundary condition was set to fluctuate freely.

Blood was considered a Newtonian fluid with dynamic viscosity *µ* = 3.5 × 10^*−*3^ Pa s and density *ρ* = 1.06 × 10^3^ kg m^*−*3^.

Since it is believed that WSS has significant influence in the patency of AVFs by its role in the genesis of intimal hyperplasia [15], both time-averaged WSS

1 $${\text{TAWSS}}\; = \;\frac{1}{T}\mathop \smallint \limits_{t0}^{t0 + T} \left\| {\tau_{\text{w}} } \right\|{\text{d}}t$$

and oscillatory shear index

2 $${\text{OSI}}\;{ = }\; \frac{1}{2}\left( {1 - \frac{{\mathop \smallint \nolimits_{t0}^{t0 + T} \left\| {\tau_{\text{w}} {\text{d}}t} \right\|}}{{\mathop \smallint \nolimits_{t0}^{t0 + T} \left\| {\tau_{\text{w}} {\text{d}}t} \right\|}}} \right)$$

were used in the analysis. In Eqs. (1) and (2), ***τ****w* is the WSS tensor, *T* is the pulse period, and *t0* is a point in time. The *OSI* shows whether the ***τ****w*$\hat{\varvec{n}}$ vector oscillates on a single pulse orientation (OSI = 0.0) or oscillating between positive and negative orientations (OSI = 0.5) during the pulse period, where $\hat{\varvec{n}}$ is the surface normal vector. Time averaged quantities were measured in the third pulse (i.e., *t*0 ≡ 2*T)* of the simulation in order to remove flow-dependent effects.

### Phantom Signal Analysis

In order to profile each imaging sequence, signal intensity measurements were taken using FIJI.^19^ Circular regions of interest (RoI) were used to measure signal from the flowing water, background and stationary water sources in the image. This was done manually for each slice in the series. Signal intensity values were normalized using a min-max normalization algorithm available in scikit- learn^17^about:blank. Signal distributions were visualized graphically using the Seaborn and matplotlib libraries in Python 2.7.

The STL model for the curved phantom was used to assess correlations between the signal intensity to the flow angle relative to the magnetic field direction in the curved phantom. Tangent unit vectors $\hat{\varvec{t}}$ = d***γ***/d*s* were sampled at intervals ∆*s* = 1 mm along the centerline. The metric

3 $$\varGamma \; = \;\hat{\varvec{t}} \cdot \hat{\varvec{B}}0$$

defined by the dot product between $\hat{\varvec{t}}$ and the B0 (z-axis) unit vector was calculated for each instance in order to assess the effect of flow-direction. Γ takes values of 0 for flow parallel to B0, and values of 1 for flow perpendicular to B0. Correlations between Γ and the signal intensity were assessed graphically, and with Spearman’s correlation statistic. Similarly, correlations between signal intensity and the first spatial derivative of Γ (i.e. the instantaneous curvature) were assessed

4 $$\varGamma^{\prime} = \frac{{{\text{d}}t}}{{{\text{d}}s}} \cdot B_{0} .$$

### Data Analysis and Comparison Methods

Agreement between diameter measurements from MEDIC and ToF phantom images was assessed using Bland-Altman methodology.^5^ For each area measurement, the mean and difference between the MEDIC and ToF model areas were calculated. This was done for the full length of the straight phantoms, and for a length of 5 cm centered on the curve center for the curved phantoms.

Results from CFD simulations were interpolated onto the same space as area measurements, allowing observation of the effect of area on WSS quantities. The mean value for all measured quantities was calculated at 1mm intervals from the origin as previously described, and plotted as a function of distance from the origin. Differences in the segmented models area and WSS quantities from CFD simulation for the patient data were interpreted graphically throughout the length of the vessels for the patient cases.

Differences in measurements were also interpreted numerically through the use of an error metric which was defined as the summed difference divided by the average:

5 $$E_{f} \; = \; \frac{2}{N}\mathop \sum \limits_{i = 1}^{n} {\text{abs}}\left( {\frac{{f_{T,i} - f_{M,i} }}{{f_{T,i} + f_{M,i} }}} \right)$$

where *n* is the number of sampled data, and *f*_T_ and *f*_M_ are any quantity *f* obtained from the ToF and MEDIC images, respectively. *E*_*f*_ takes a value of zero for identical measurements, and increases as the difference between the measurements increases.

## Results

### Phantom Analysis

A number of observations were common to all phantom cases:

- The signal intensity from flowing water was similar for both sequences.
- Signal intensity from the water used to prime the scanner was higher on the MEDIC than the ToF.
- Background noise was higher for the ToF image series.

**Figure 1:**
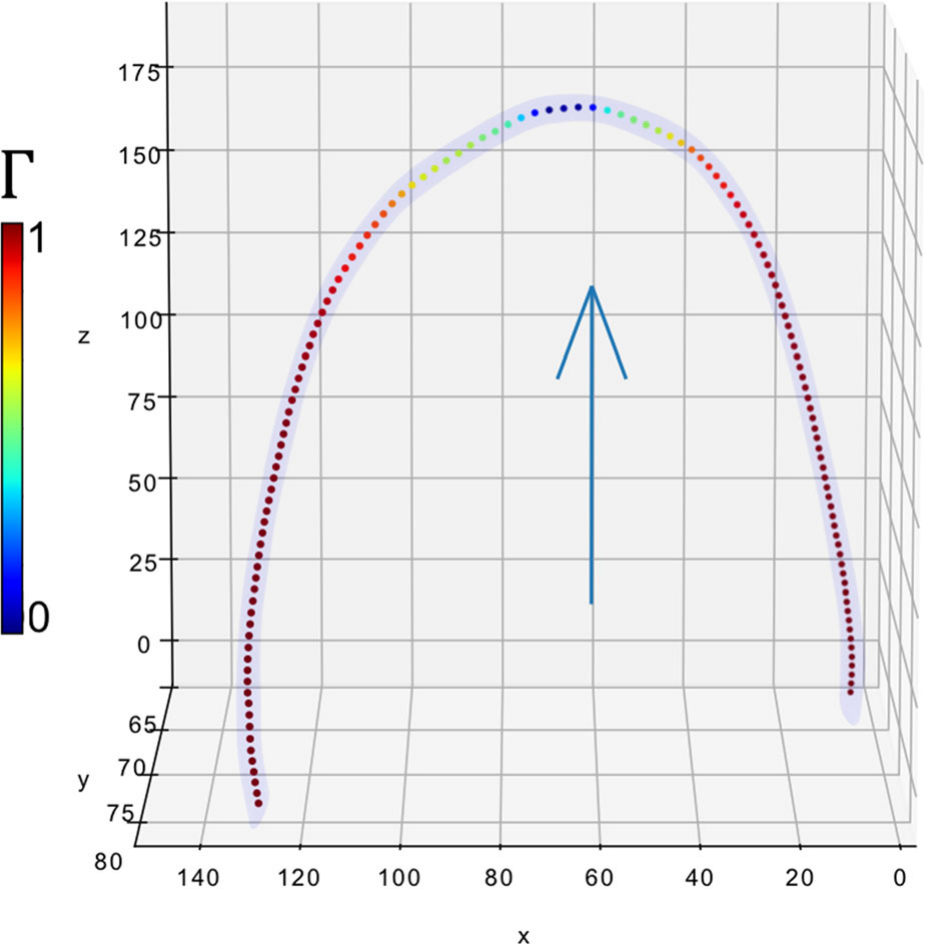
3D visualization of curve-H phantom, showing the sampled points along the vessel colour-coded to the values of Γ. As expected, Γ takes a value of zero when flow is at a right angle to B0 (blue arrow).

**Figure 2:**
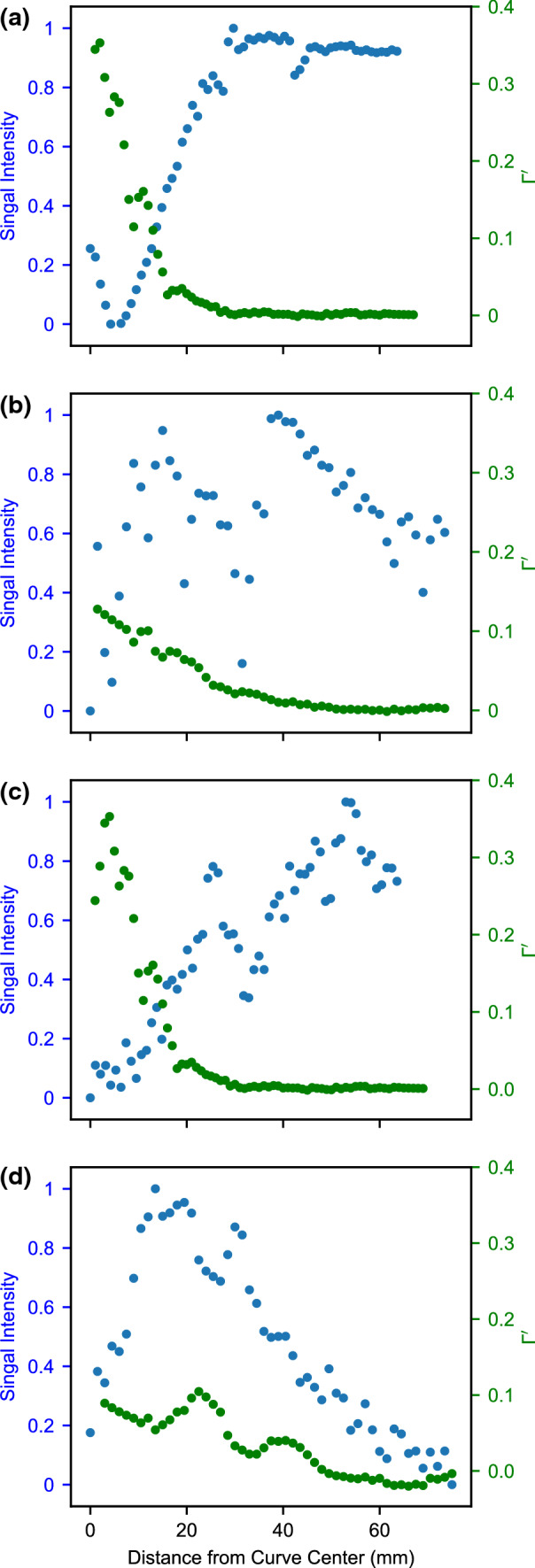
Signal dependency on rate of change of flow direction for: (a) curve-H MEDIC, *r* = − 0.85, *p <* 0.05; (b) curve-H ToF, *r* = − 0.85, *p <* 0.05; (c) curve-L MEDIC, *r* = − 0.89, *p <* 0.05; (d) curve-L ToF, *r* = 0.47, *p <* 0.05.

Example MRI images of the phantoms can be seen in the supplementary material (sup. Fig. 4). Flow direction data were generated as described, and are visualized in Fig. 1. Signal intensity was plotted alongside **Γ**, and no clear trend was observed (data not shown). When the signal intensity was plotted alongside the first derivative, **Γ**^***′***^, a correlation was observed with the MEDIC sequence. This is visualised in Fig. 2. The signal intensity of the MEDIC exhibited a strong negative correlation with **Γ**^***′***^, for both phantom geometries, as assessed with Spearman’s correlation coefficient (curve-*L*, *r* = −0.89, *p <* 0.05; curve-*H*, *r* = −0.85, *p <* 0.05). Phantom curve-H lost all visible signal near the curve centre. No such observation was observed consistently on the ToF images.

**Figure 3:**
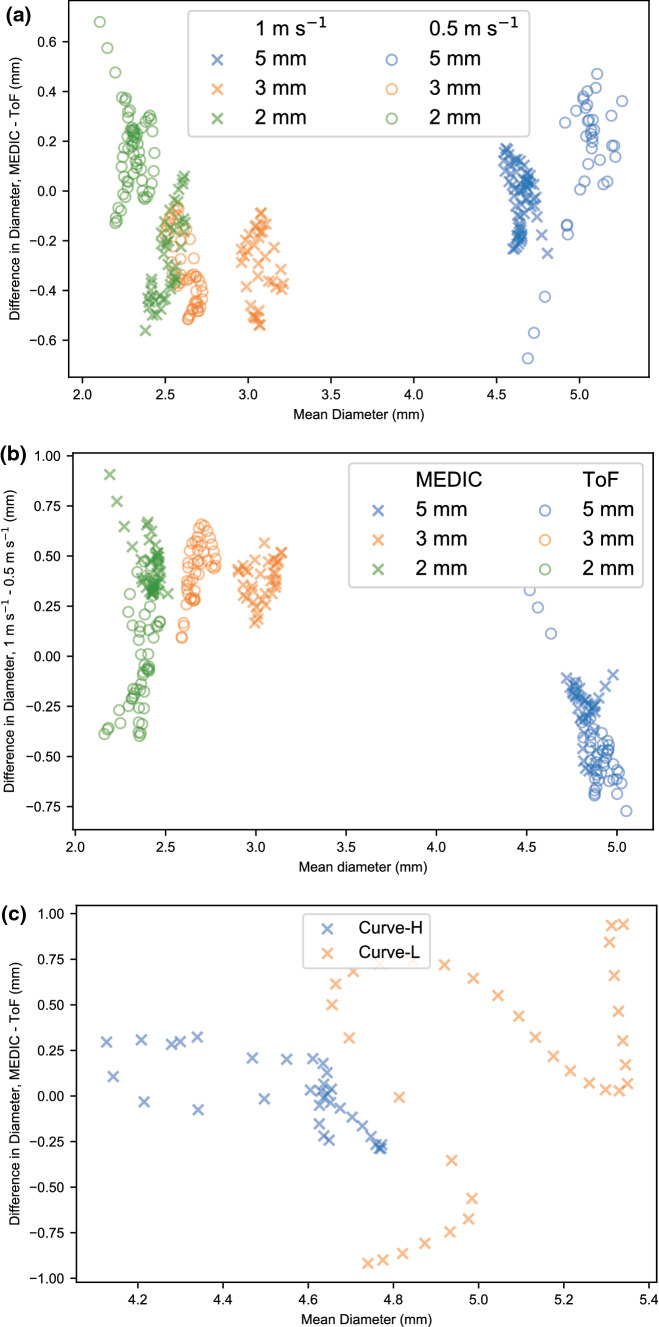
Bland–Altman plots for all phantom cases, showing differences in the diameter measurement on the y-axis, and mean value on the x-axis. (a) Agreement between MEDIC and ToF sequences for different flow and diameter rates; (b) agreement between high and low flow for different diameter and MRI sequence; (c) agreement between high and low-curve, with diameter = 5 *mm* and flow velocity = 1 m s^−1^.

Values for *E*_*A*_, along with mean differences in diameters for the phantom cases are reported in Table 1. Values of *E*_WSS_ were higher than *E*_*A*_, with error largest in the 3 mm phantom, which was possibly caused by a misalignment or kink in the tubing. From Table 1, mean differences in diameter for the phantoms can be seen to be around 0–15% for all cases. Differences between the imaging sequences increased as the vessel radius decreased, however no other clear trend was observed. Bland-Altman plots for these phantom geometries can be seen in Fig. 3. A larger degree of variability was observed when the flow-velocity was altered as can be visualised in Fig. 3(b). For the curved phantoms, the largest deviation was seen at the center of the curve. Bland-Altman plots for these phantom geometries can be seen in Fig. 3(c).

**Table 1:** Error analysis between MEDIC and ToF sequences for phantom STL and CFD measurements at a flow velocity of 1 m s^−1^.

Phantom	E_A_ (−)	E_wss_ (−)	Mean difference in diameter (mm)
Straight, 5 mm	0.04	0.28	− 0.02 ± 0.12
Straight, 3 mm	0.20	0.47	− 0.3 ± 0.14
Straight, 2 mm	0.22	0.29	− 0.26 ± 0.16
Curved-*H*, 5 mm	0.03	–	0.00 ± 0.20
Curved-*L*, 5 mm	0.10	–	0.20 ± 0.60

### Patient Analysis

**Figure 4:**
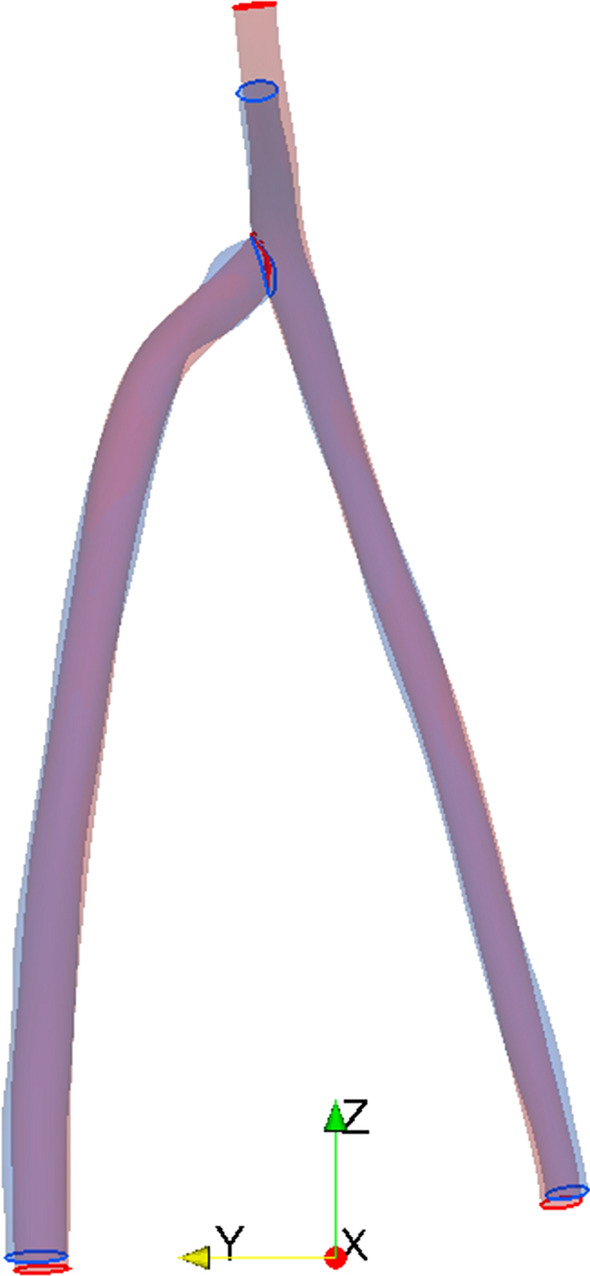
Overlapping segmentations of the MEDIC (blue) and ToF (red) images of patient AVF1.

**Figure 5:**
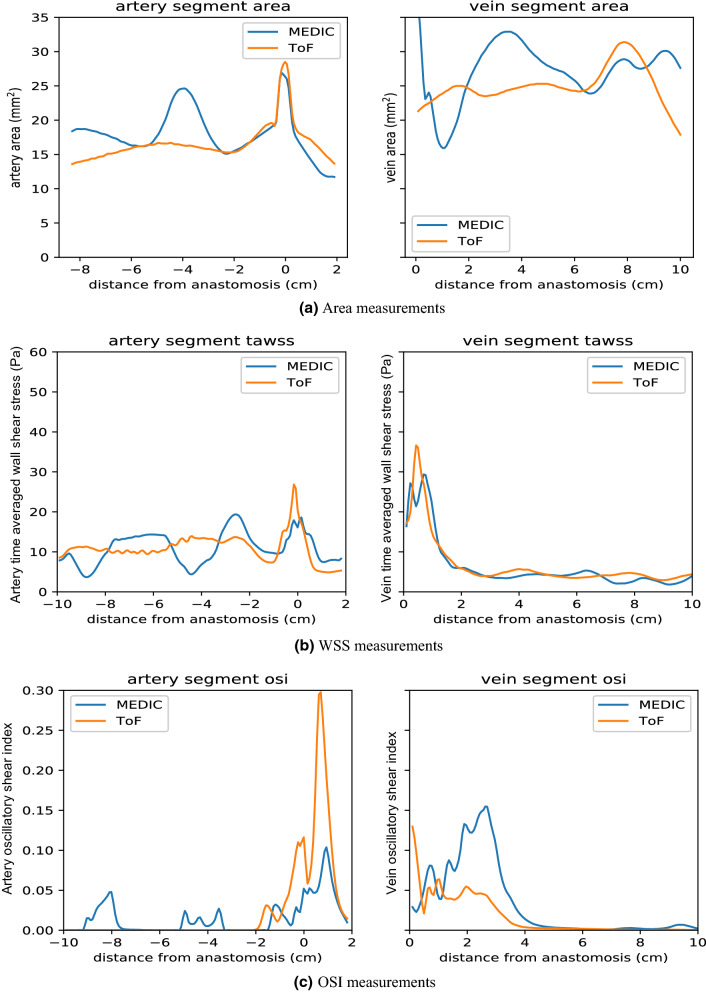
(a) Artery and vein area measurements with origin defined at the anas tomosis; (b) WSS measurements; (c) OSI measurements for patient AV1.

Segmentation using both MEDIC and ToF sequences was possible for all MRI series. Examples of the patient MRI images can be seen in the supplementary material (supp. Fig. 5). Nevertheless, the 3D segmentations of the same patients were not completely identical when comparing the MEDIC and ToF sequences. The segmentation end points were at different positions, because the FoV of the two sequences were not over a precisely shared volume (see Fig. 4). For example, the depiction of vessel area was generally larger for the MEDIC, although the lumen area, shape and curvature were variable throughout the length of all vessels studied. For instance, the venous section of the MEDIC sequence of patient AVF1 revealed a larger anastomosis, a narrowing around 1 cm, as well as a dilation around 4 cm from the anastomosis (see Fig. 5(a)). Signal drop-out was observed in the area near the anastomosis, meaning that segmentation was reliant on interpretation at this point. All these variations impact the flow dynamics in the CFD study.

**Figure 6:**
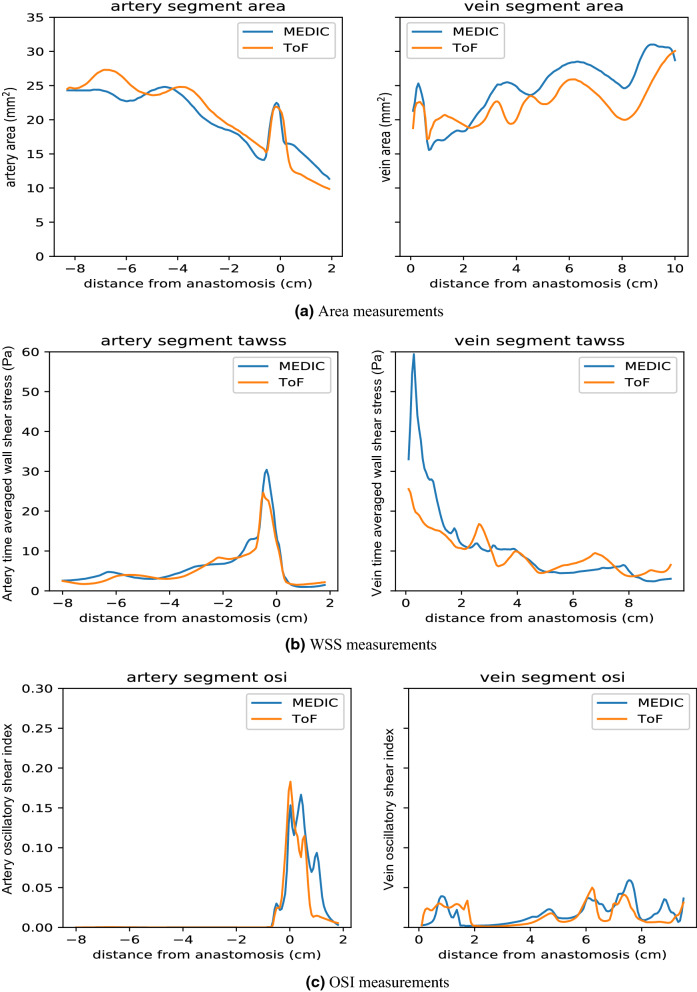
(a) Artery and vein area measurements with origin defined at the anas tomosis; (b) WSS measurements; (c) OSI measurements for patient AV2.

Area variability directly interferes with the hydrodynamics of internal flows, but the order of this influence depends on other flow features too. By looking at Figs. 5 and 6, one can see that area and TAWSS have a negative correlation at many points, but not in the whole extent of the vessels. The negative correlation between area and WSS is observed in the arterial segment of the MEDIC sequence, where flow was mostly unidirectional. However, this negative correlation is less apparent in the venous segment nearer the anastomosis, due to the presence of more complex flow patterns, such as flow separation and jet oscillations. These flow features redistribute the velocity gradients away from the vessel wall, minimizing the sensitivity of TAWSS to cross-sectional area variations.

**Figure 7:**
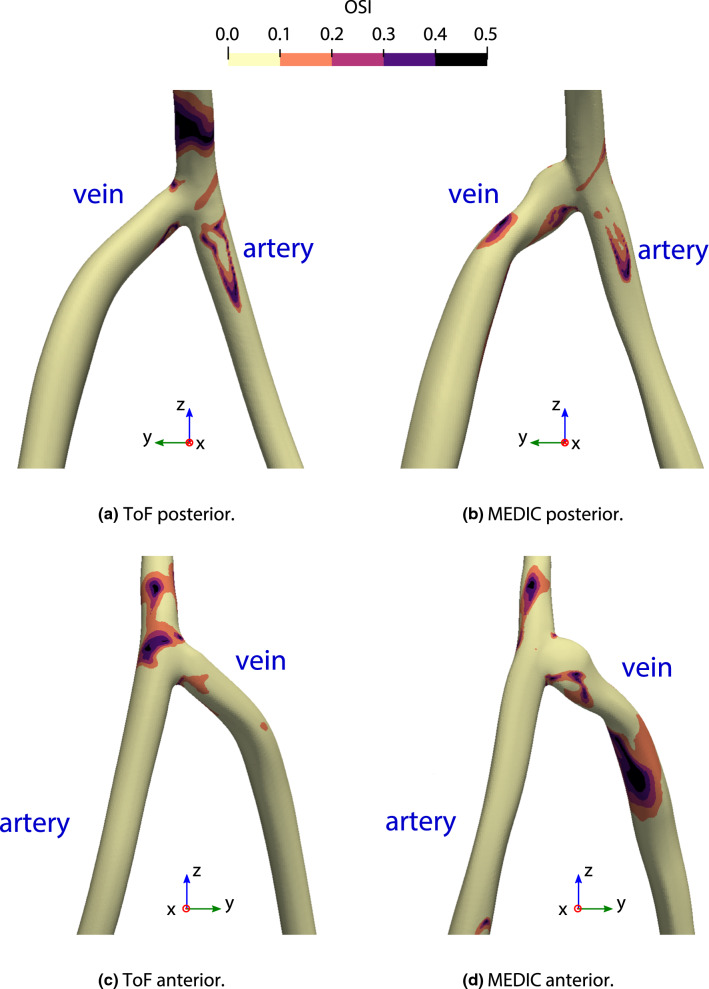
Oscillatory shear index of the third pulse of patient AV1.

**Figure 8:**
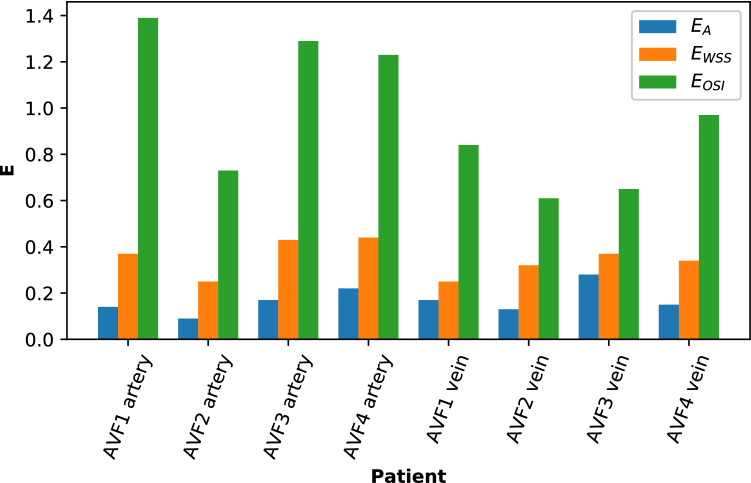
Error analysis for patient STL and CFD measurements.

The OSI shows how strongly the direction of WSS changes during the pulse cycle due to flow reversal or varying flow direction. In general, the regions of high OSI do not correlate with regions of high TAWSS. In Fig. 7, it is possible to compare the spatial distribution of OSI for both MEDIC and ToF sequences of patient AVF1. *E*_*f*_ values for all patient cases, including area, WSS and OSI can be seen in Table 2 and Fig. 8.

**Table 2:** Error analysis between MEDIC and ToF sequences for patient STL and CFD measurements.

Vessel	Patient	E_A_	E_TAWSS_	E_OSI_
Artery	AVF1	0.14	0.37	1.39
	AVF2	0.09	0.25	0.73
	AVF3	0.17	0.43	1.29
	AVF4	0.22	0.44	1.23
Vein	AVF1	0.17	0.25	0.84
	AVF2	0.13	0.32	0.61
	AVF3	0.28	0.37	0.65
	AVF4	0.15	0.34	0.97

## Discussion

This study has demonstrated that the choice of MRI sequence can lead to subtle differences in segmented model geometry and corresponding CFD results. The differences are of varying magnitude throughout the length of the vessel, suggesting that local effects are causing the observed differences. The effect of this area variation caused disagreement between the CFD results from the two MRI sequences.

We analyzed the signal distributions of the MEDIC and ToF sequences using a series of phantoms in an attempt to determine the cause of area variations, and observed no differences in the signal intensity profiles from the vessels. Plots of signal intensity revealed the ToF sequence was noisier, and the MEDIC produced a higher signal from stationary water used to prime the scanner, as expected. We then created an analogous geometry to the curved swing-site segment of the AVF using two curved phantoms and analyzed the effect of an off-axis flow on signal intensity measurements for two different curvatures. This led to the observation that for the MEDIC sequence, the signal intensity was inversely correlated with the instantaneous curvature (as assessed by **Γ**′), which may be a contributing factor in the observed signal dropout in the patient images. Signal loss in the curved phantom was not apparent on the ToF images, but both sequences exhibited signal loss on the patient images at the swing-site, suggesting that local flow effects at the anastomosis may have more effect than flow-direction effects as represented by **Γ** (Eq. 4).

CFD has been widely used in the study of AVFs. Multiple studies have assessed flow distributions in AVFs, using patient specific models, with imaging modalities such as 3D ultrasound, MRI, and CT having been used to obtain patient specific data. Considering MRI, multiple sequences such as T2 weighting, black-blood, and ToF have been used. As our results indicate, the distributions of flow parameters utilizing spatial data from different modalities may not be comparable. For example, McGah *et al*.^14^ used 3D US to create patient specific models, and observed increased WSS around the anastomosis, and also in the proximal vein of an AVF. Using BB-MRI, He *et al*.^11^ observed increased WSS at only the anastomosis. Sigovan *et al*.^20^ used a 2D ToF sequence and identified increased WSS again at the anastomosis, but also in the proximal vein and the distal artery. Similarly, Ende-lordache *et al*.^10^ identified increased WSS at the anastomosis, and also increased OSI in the venous and arterial segments of the AVF. As demonstrated in our work, we cannot yet be sure which model could be considered the most accurate of these cases. One reassuring aspect is that the distributions are generally similar, and the anastomosis is consistently cited as an area of increased WSS. We can be relatively confident that the anastomosis undergoes high WSS, despite being unsure of the magnitude, or other specific locations.

The MEDIC sequence has been observed to give vessel diameter measurements in agreement with US in the upper periphery, before and after AVF creation.^13^ The area disagreement *E*_*A*_ between the straight phantoms increased as the tube diameter was decreased, which would be expected as the size of the phantom reduces and approaches the resolution of the scanner. However, the mean difference was consistently maximised at around 15% in the straight phantoms, similar to the error reported in diameter in other studies of vessels used for AVF creation.^18^ The differences in error between the flow velocities assessed demonstrate how one a sequence which may be working well for a flow of 0.5 m s ^−1^ may perform poorly with higher or lower velocities, and vice-versa. The error was also increased in the curved phantoms as compared with the straight phantoms of the same diameter. Error in WSS was typically lower in the phantoms than in the patient cases, which is reassuring given that the vessels used were lower in diameter than we would expect from matured AVF vessels.

The distributions of WSS and OSI in the patient models were observed to differ between the sequences. As the only difference in the simulations was the vessel geometry, it is evident how sensitive WSS/OSI are to variations in geometry. Intermittent flow separations occurred at different positions, such as on the posterior and anterior vessel walls of the MEDIC and ToF sequences respectively. In general, MEDIC showed more regions of high OSI than ToF, because the geometry from MEDIC had more variability in cross-sectional area than ToF due to its lower slice thickness. In light of these results, further study should be undertaken to determine which sequence produces geometries which agree with experimental results.

The anastomosis of the AVF introduces challenges for MR imaging, and signal drop-out was observed in all the anastomoses studied. Flow recirculation at the anastomosis is reported in simulations of AVFs,^9^ and it is likely that this effect could be a major cause of spin dephasing^24^ resulting in signal loss at the anastomosis. Due to the orientation of the swing site, flow is no longer confined along the z-axis of the B0 field, effectively reducing the velocity of any incoming spins relative to the B0 field, which can be another cause of signal loss. AVF flow is known to be turbulent, and this is the cause of the signature ‘thrill’ of the AVF. Turbulent flow can undergo spin dephasing causing MR signal heterogeneities, however, turbulent flow was observed in the phantoms, and did not seem to decrease the MR signal. Further, due to the nature of the anatomy, the region of interest (the arm) is typically placed in a region of heterogeneous magnetic field. These problems may act together to lower signal at the swing site and anastomosis, making this region particularly difficult to segment and heavily reliant on operator interpretation, an under-reported aspect in most CFD based studies of AVFs.

These geometric errors propagate into larger errors for parameters derived from CFD. The error metric *E*_WSS_ was larger than *E*_*A*_ for every phantom and patient vessel studied (tables 1, 2 and Fig. 8). Wall shear stress and cross-sectional area are inversely related through a straight vessel, as given by the Darcy-Weisbach equation

6 $$\tau w = \frac{{f_{\text{D}} }}{8}\rho \left( {\frac{Q}{A}} \right)^{2}$$

where *f*_D_ is an empirical friction factor, *ρ* is fluid density, *Q* is flow rate, and *A* is area. In the laminar regime, the relation of WSS and area is weaker (*τw* ~ *A*^*−*1.5^), because the friction factor is inversely proportional to the Reynolds number (*Re*), *f*_D_ = 64/*Re*. However, in the turbulent regime, the friction factor does not scale with *Re*, but it rather depends more on the roughness of the vessel, which leads to a second-order inverse relationship between WSS and area (*τw* ~ *A*^*−*2^). Hence, it is important that accurate measurements of vessel area are obtained for accurate estimation of WSS. As OSI is derived from WSS, and depends on more non-linear effects, such as flow separations, *E*_OSI_ was larger than *E*_*A*_ and *E*_WSS_ for all cases studied.

This study has a number of limitations. The Reynolds number of the phantoms was typically higher than we would expect from AVFs, and despite the flow-rates being in the optimal region for haemodialysis, real vessels would ideally be at least 5mm. The slice thicknesses of the sequences were not identical, however the voxel sizes were similar. Reducing the slice thickness on ToF resulted in unacceptable SNR losses. A major advantage of the MEDIC is the ability to acquire thinner slices with superior SNR to even the thicker ToF. Despite both sequences suffering from signal loss around the anastomosis, the MEDIC sequence does provide good vessel edge detail at the locations in and around the anastomosis^13^ facilitating segmentation, and it is possible that this may prove useful as a surveillance tool to characterise the development of stationary structures such as possible stenosis sites. One user segmented the geometries from all MR image sets, so it was not possible to assess variability between operators. This is well covered in the literature and was not the purpose of our study. We did not compare our results to an experimental model, such as in the FDA-sponsored study.^21^

Importantly, we did not use specifically optimized sequences for imaging of the different flow velocities studied. Optimization of the sequences could have been performed to find the parameters giving the truest geometric depiction for each flow velocity, or to identify the parameters which reduce the error between the velocities. However, as we were imaging both arteries and veins with different flow velocities in one sitting, optimization would have resulted in a trade-off between venous or arterial depiction, thus we opted to use vendor default parameters. If researchers could optimize sequence parameters prior to imaging, this would be one method to increase geometric accuracy before progressing to CFD. Similarly, the boundary conditions used for CFD modeling could be further optimized. Whilst we made the assumption that the outflow would be of a parabolic distribution at a distance of 5 cm from the anastomosis, this could be considered a simplified case. It is possible that more complex flow patterns may be present distal from the anastomosis. Different boundary conditions could be considered in advances in this line of research.

No comparison was made with any black-blood sequences or other modalities such as CT, which are also used to create 3D models for CFD studies of AVFs. Black-blood MRI has been shown to yield good results when measuring vessel parameters for intracranial vessels.^4^ Similarly to this work, other authors signify that sequence parameters affect wall measurements and sharpness of the vessel wall borders, which require optimization prior to commencing the study. Black blood MRA could in theory help with measurements of rapidly flowing or turbulent blood, which may yield low signal due to loss of spins phase-coherence and in-flow effects. Use of black-blood techniques in lumen measurements of AVFs and CFD modeling of wall shear stress has been demonstrated.^11^ However, 3D black-blood scans take longer to perform,^12^ resulting in scan times that can be uncomfortable for some patients.

Another emerging method not considered in this study is 4D flow MRI. In addition to morphological information, this technique has been shown to non-invasively characterize physiological properties, such as velocity, flow volume, wall shear stress, pressure gradients, streamlines and flow path lines in cerebral arterio-venous malformations.^8^ 4D flow MRI has been shown to currently underestimate WSS values in intracranial arteries, but with good estimate of WSS distribution when compared to CFD methods.^22^ As 4D flow methods would remove the need for segmentation, any error associated with this stage could be eliminated. However, one limitation of this technique is the long scan time required, which may be a difficulty for certain patients. A number of studies have also reported underestimation of 4D flow derived velocities when compared to Doppler US, which would need to be considered when assessing flow properties.^15^, ^27^

In conclusion, we have demonstrated that different MRI sequences do not give reproducible results when considering CFD studies of AVFs. Small geometric differences encountered during imaging propagated into larger differences during CFD modeling, meaning results were not fully comparable between MRI sequences. Vessel diameter, flow velocity, and patient specific flows were all sources of error that could have caused these differences. The results from this study should be taken into consideration when planning patient specific CFD studies and researchers should justify their choice of MRI sequence *a-priori*. Comparisons with known results from experiment should be performed to fully understand the impact of changing MRI sequence, and to determine which sequences provide geometries closest to the ground-truth.

## Electronic supplementary material

Below is the link to the electronic supplementary material.

Supplementary material 1 (PPTX 1903 kb)
