# Supplementary material for: Experimental Assessment of Two Non-Contrast MRI Sequences Used for Computational Fluid Dynamics: Investigation of Consistency Between Techniques
Source: Cardiovasc Eng Technol. 2020 Jul 1;11(4):416–30. doi: 10.1007/s13239-020-00473-z (PMC7385008; doi:10.1007/s13239-020-00473-z)
Supplement: Supplementary file 1 — Supplementary material 1 (PPTX 1903 kb) [file 13239_2020_473_MOESM1_ESM.pptx]

## Slide 1
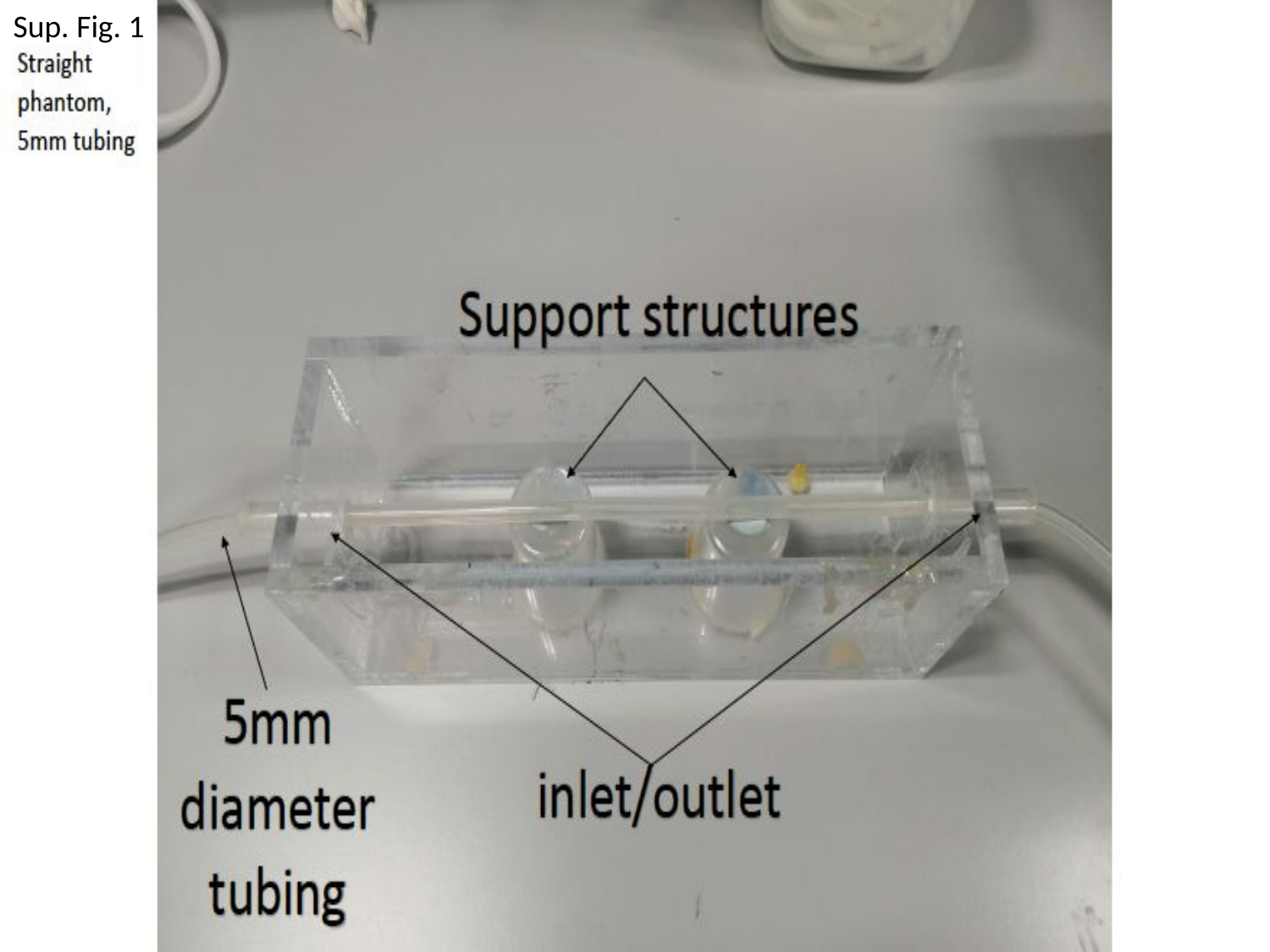

Sup. Fig. 1
#

## Slide 2
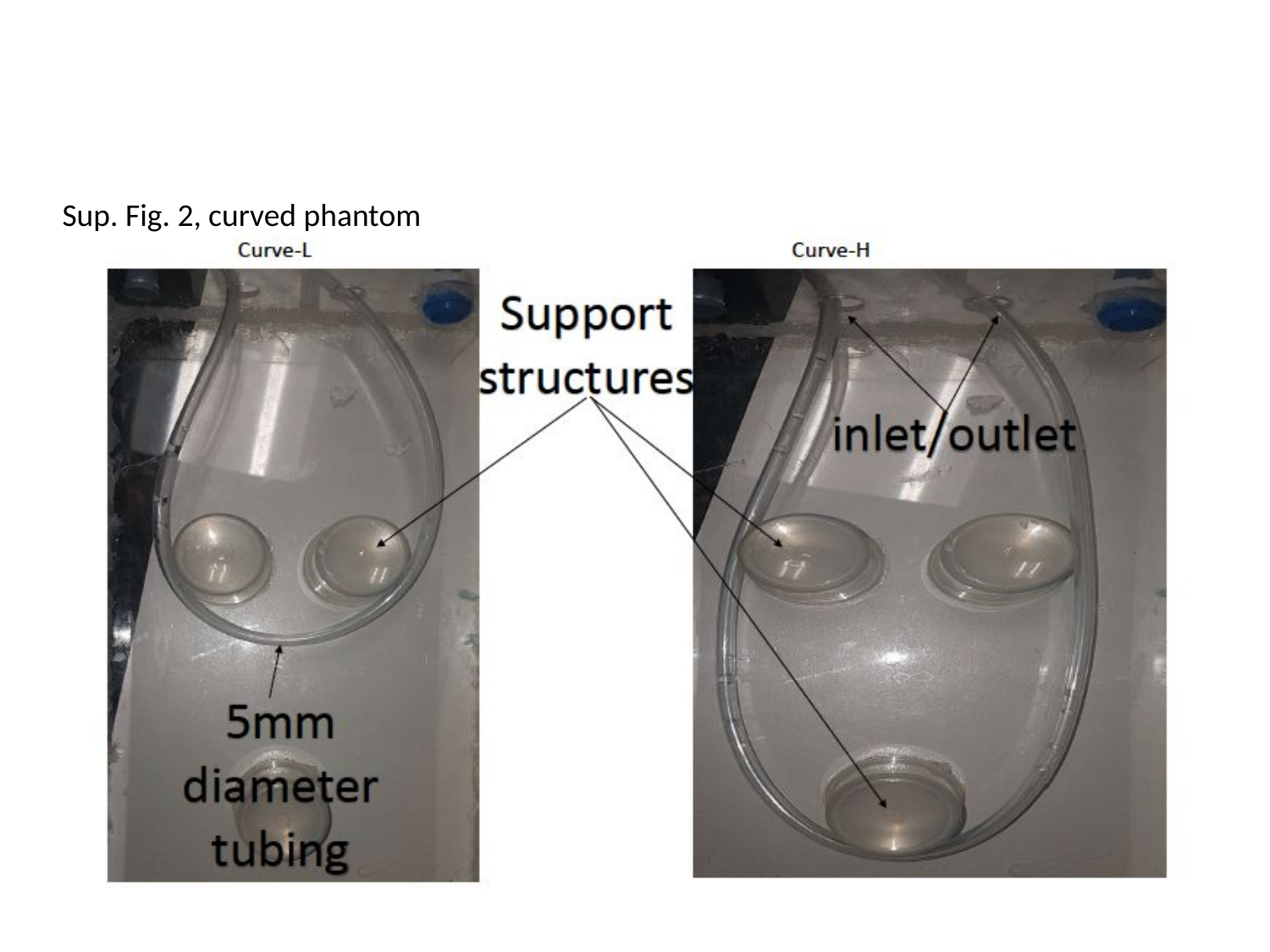

Sup. Fig. 2, curved phantom

## Slide 3
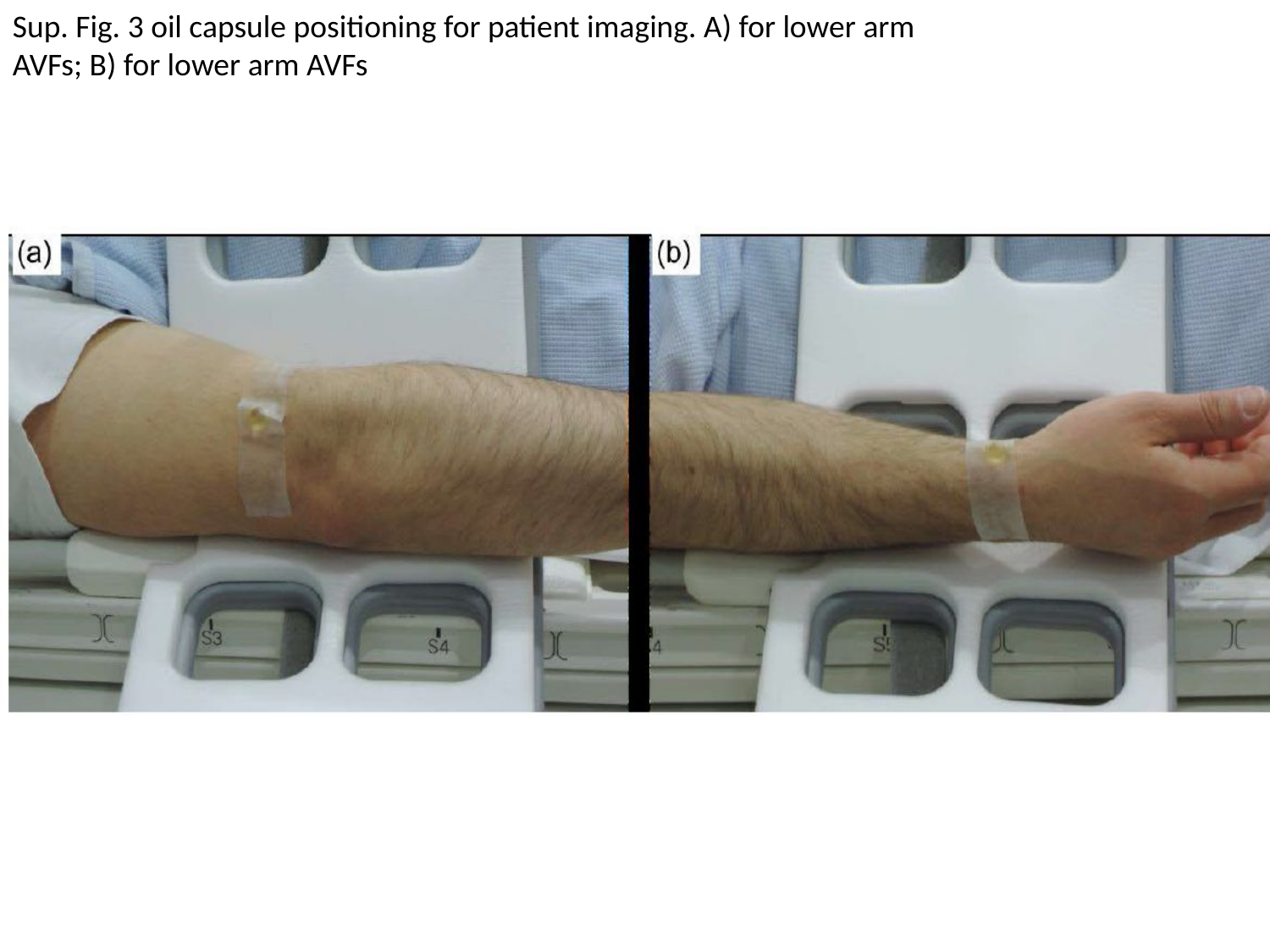

Sup. Fig. 3 oil capsule positioning for patient imaging. A) for lower arm AVFs; B) for lower arm AVFs

## Slide 4
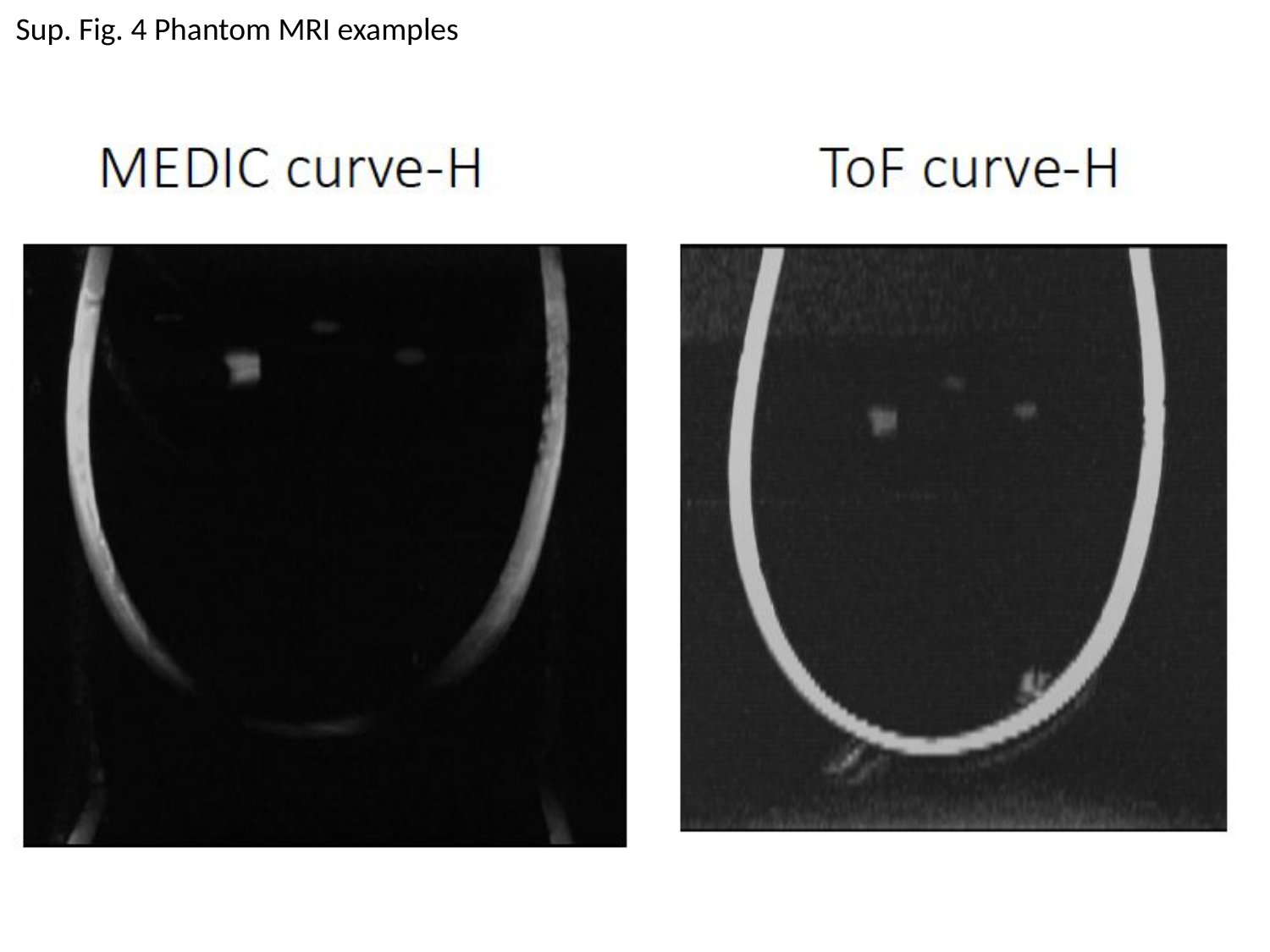

Sup. Fig. 4 Phantom MRI examples

## Slide 5
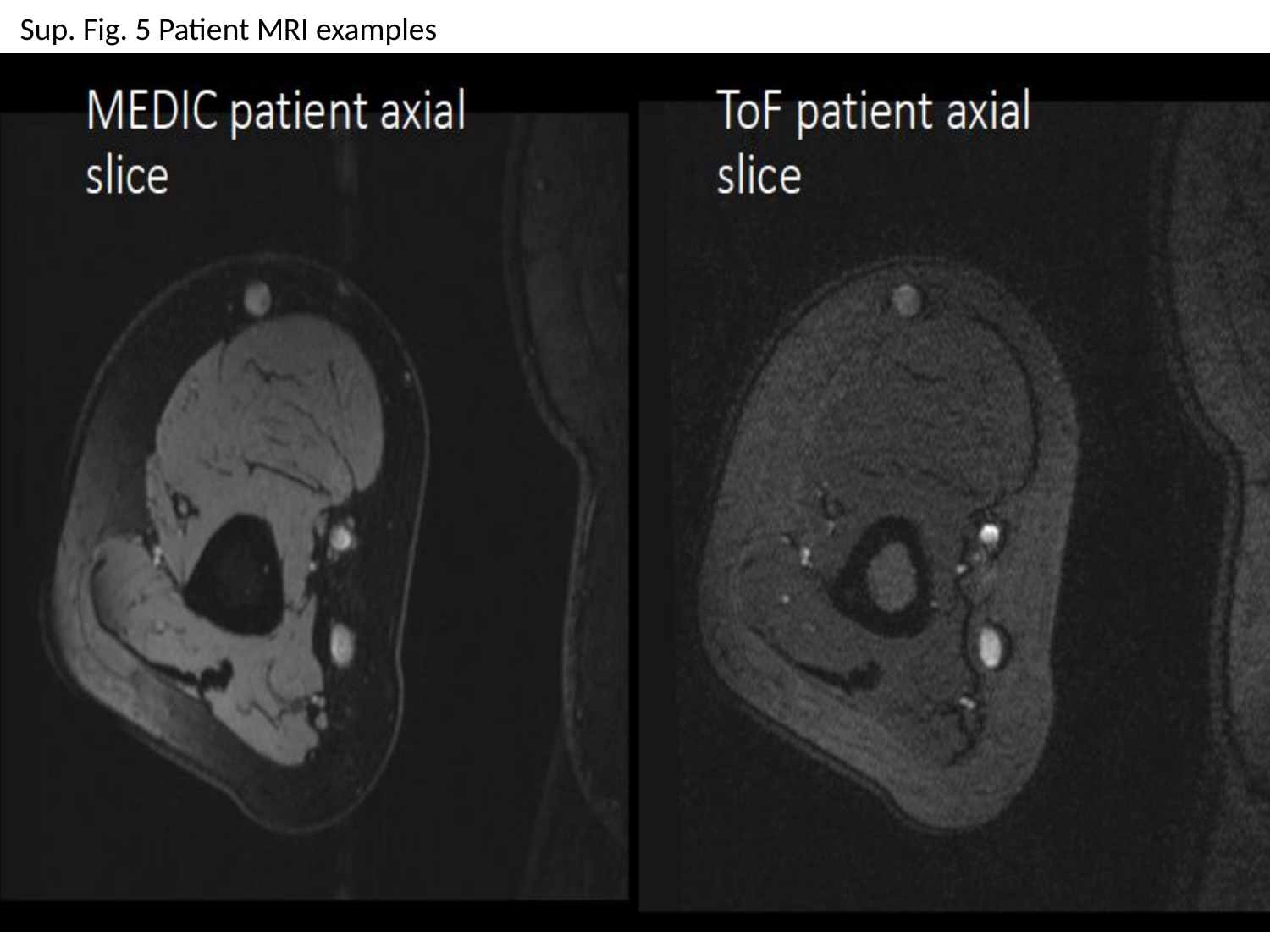

Sup. Fig. 5 Patient MRI examples
